# Comparative Efficacy of Transurethral Laser Ablation Versus Conventional Methods in the Management of Recurrent Non-Muscle-Invasive Bladder Tumours

**DOI:** 10.7759/cureus.94455

**Published:** 2025-10-13

**Authors:** Mahmoud D Srour, Ali Mohamed, Sunil Mathur

**Affiliations:** 1 Urology, Great Western Hospitals NHS Foundation Trust, Swindon, GBR

**Keywords:** non-muscle-invasive bladder cancer, outpatient tula service, recurrent superficial bladder tumours, transurethral laser ablation (tula), transurethral resection of bladder tumour (turbt)

## Abstract

Introduction: Non-muscle-invasive bladder cancer (NMIBC) is characterised by a high recurrence rate, necessitating frequent intervention. While conventional transurethral resection of bladder tumour (TURBT) under general anaesthesia is standard, it carries inherent risks. Transurethral laser ablation (TULA) performed under local anaesthesia in an outpatient setting presents a viable, minimally invasive alternative, yet robust comparative efficacy data remain limited. This study aimed to compare the effectiveness of TULA and TURBT in preventing early tumour recurrence and to provide practical insights for implementing an outpatient TULA service.

Methods: This retrospective cohort study included patients with recurrent NMIBC treated with either TULA or TURBT. Patient selection for the comparative analysis was restricted to those with recurrent lesions sized less than 1 cm. The TULA procedures were performed under local anaesthesia using a holmium:yttrium-aluminium-garnet (Ho:YAG) laser (Cook Medical, Ireland) and a compatible fibre. Key outcomes included the tumour recurrence rate at the first follow-up cystoscopy. Complications were assessed retrospectively over a 30-day period.

Results: A total of 97 patients were included (TULA: 51; TURBT: 46). Baseline characteristics (age, sex, and risk stratification) were comparable between the groups (p>0.05). No statistically significant difference in tumour recurrence rates was observed at the first follow-up cystoscopy: 30 patients (58.8%) in the TULA group and 27 patients (58.7%) in the TURBT group exhibited no recurrence. Furthermore, TULA demonstrated no immediate post-procedure complications requiring hospital admission. Implementation of the outpatient TULA service, prioritising staff training, safety, and a robust governance framework, proved successful in our centre.

Conclusion: TULA performed under local anaesthesia demonstrates possible comparable efficacy to TURBT in preventing early tumour recurrence, underscoring its potential as a reasonable alternative for managing small, recurrent NMIBC, particularly for patients at higher risk of complications from general anaesthesia. Successful establishment of an outpatient TULA service, guided by clear operational and safety protocols, enhances efficiency. Larger prospective multicentre trials with longer follow-up are warranted to fully validate these findings.

## Introduction

Recurrent non-muscle-invasive bladder cancer (NMIBC) represents a common urological concern with a recurrence rate of 31-78% of cases within five years [1]. The highest risk is observed in patients with high-grade or multifocal disease, while the lowest risk is seen in solitary, low-grade tumours. This situation necessitates frequent interventions to prevent progression to invasive disease. Historically, transurethral resection of bladder tumour (TURBT) or cystodiathermy has been the cornerstone of treatment, albeit with inherent risks associated with general anaesthesia. The emergence of transurethral laser ablation (TULA), performed under local anaesthesia, presents a compelling alternative, potentially mitigating anaesthesia-related risks while maintaining oncological efficacy [2,3].

Previous studies and meta-analyses have demonstrated comparable oncological outcomes between TURBT and laser-based approaches, with potential advantages in terms of complication rates - up to 43.5% of cases experienced at least one complication within 30 days following TURBT [4] - and cost-effectiveness. However, there are limitations in prior research, including a lack of extensive studies on this emerging technology, small sample sizes, shorter follow-up periods, and selection bias [4-7]. This study aimed to assess the comparative efficacy of TULA versus TURBT in preventing tumour recurrence and to outline the implementation process of an outpatient TULA service in a growing urology centre.

## Materials and methods

A retrospective cohort study was conducted using data from patients who underwent either TULA or TURBT between July 2020 and December 2021 at Great Western Hospitals NHS Foundation Trust in Swindon, United Kingdom (UK). A total of 97 patients were included, with 51 in the TULA group and 46 in the TURBT group. All patients had a recurrence of a low- to intermediate-risk primary tumour, which was less than 1 cm, except two patients who underwent TULA and had a primary high-risk urothelial cancer. 

**Figure 1:**
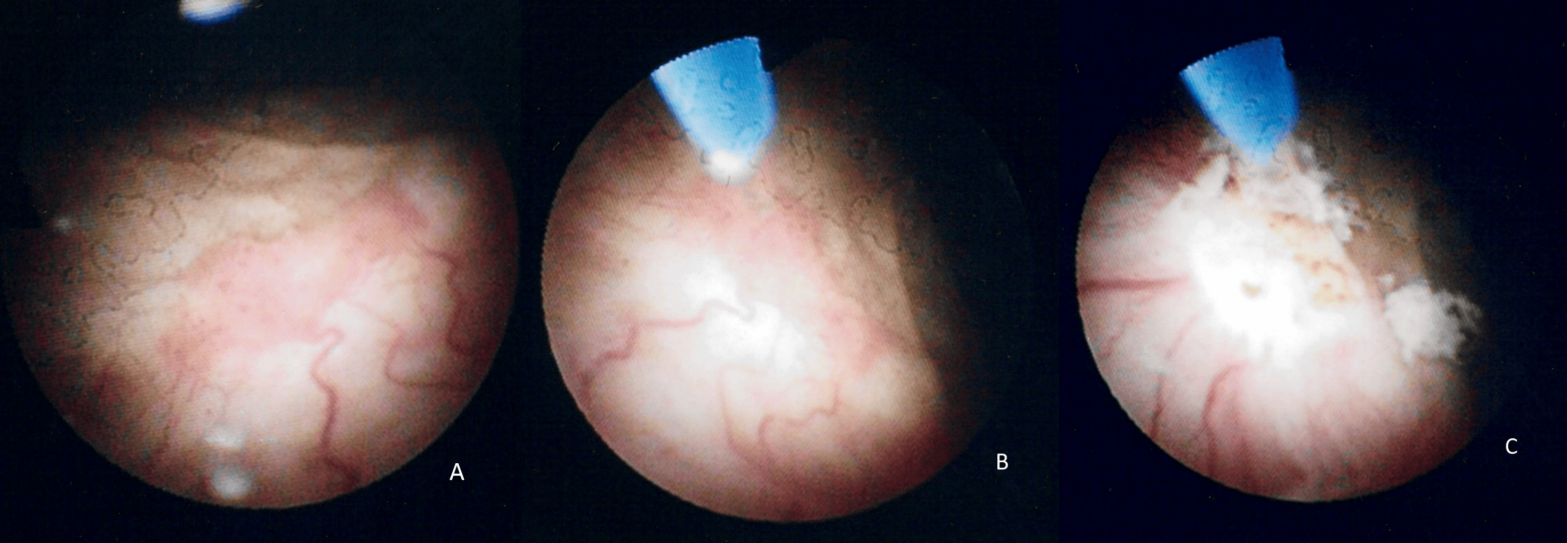
Cystoscopic management of recurrent bladder cancer with laser ablation (A) Cystoscopic view of a small recurrent bladder tumour. (B) Intraoperative positioning of the diode laser probe targeting the lesion. (C) Post-ablation appearance of the treated area, demonstrating successful tumour eradication.

All patients were treated in an outpatient setting with a 60W holmium:yttrium-aluminium-garnet (Ho:YAG) laser (Cook Medical, Ireland) [3,6]. We performed a urine dip to ensure that they were not suffering from a urinary tract infection. They did not require any prophylactic antibiotics before the procedure; no analgesia was required pre- or post-procedure [8]. The procedure itself was carried out in an aseptic manner. Prior to cystoscopy, 10 mL of Instillagel was administered. Thereafter, a 16.5 F flexible cystoscope (Olympus, UK) was used to assess the location of all tumours. Enhanced cystoscopic techniques (e.g., narrow band imaging and blue light cystoscopy) were not used. The Ho:YAG laser was then used to ablate any recurrent tumours, with a 365 nm or 200 nm compatible fibre, at 0.5 J energy and rates of 10 Hz. Normal saline solution was used as irrigation fluid through a three-way cystoscopy tap (Aquilant, UK) to allow easier irrigation if needed. We laser ablated the surface of the tumour first, followed by the base, to decrease the risk of detachment of viable tumour cells and reseeding. Biopsies of papillary recurrences are not usually taken, although they can be obtained before ablation if indicated (Figure 1).

Complications were retrospectively defined and assessed as any adverse event occurring within 30 days of the procedure, classified using the Clavien-Dindo grading system [9] - specifically Grades II-V, or Grade I complications requiring readmission. We gave patients the contact details of our endoscopy unit team to report any complications or adverse effects. We actively assessed and documented the absence of key immediate post-procedure events, including urinary tract infection, bladder perforation, and persistent haematuria requiring hospital admission. All patients underwent initial follow-up in three months after laser ablation, with subsequent cystoscopic follow-up based on the European Association of Urology (EAU) guideline recommendations, depending on their risk stratification [7]. Following the completion of this retrospective study, we started using our portable diode laser machine (CeramOptec GmbH, Germany). We use the standard machine settings for ablation (4 W at 1470 nm or 0 W at 980 nm). For red spots, we use 2 W at 1470 nm or 0 W at 980 nm settings. If there is a difficult bleeder, we use 2 W at 980 nm and 4 W at 1470 nm dual-wavelength laser settings.

Recurrence rates were evaluated during the first follow-up cystoscopy post-procedure. Data were analysed using chi-square tests for categorical variables and independent t-tests for continuous variables. All p-values were two-tailed, with a significance threshold of p<0.05. Results are presented as mean ± standard deviation (SD) for continuous variables and number (percentage) for categorical variables. Test statistics (e.g., χ², t) were reported alongside p-values. Additionally, the implementation of an outpatient TULA service was audited to ensure compliance with safety standards and procedural efficacy.

## Results

Among patients undergoing TULA, 30 patients (58.8%) exhibited no recurrence during the first follow-up cystoscopy, compared to 27 patients (58.7%) in the TURBT group. No statistically significant association between procedure type and recurrence was observed (χ²=0.001, p=0.981). There was no reporting of any unexpected post-procedure complications or adverse effects. None of the patients required readmission within the first month.

Table 1 summarises the baseline demographics of both groups. All patients had tumour recurrence size less than 1 cm. No statistically significant differences were observed in age, sex, risk stratification, or prior recurrence burden between the TULA and TURBT groups (all p>0.05). Table 2 compares recurrence rates between the two groups at first follow-up, showing no significant difference.

**Table 1:** Demographic characteristics of the patients included in the study Data are presented as mean ± SD or n (%). Comparisons between groups were made using independent t-tests for continuous variables and chi-square (χ²) tests for categorical variables. A p-value of <0.05 was considered statistically significant. TURBT: Transurethral resection of bladder tumour; TULA: Transurethral laser ablation; SD: Standard deviation

Variable		TULA (n = 51)	TURBT (n = 46)	Total (n = 97)	Test statistic	P-value
Age (years), mean ± SD		72.4 ± 8.9	71.1 ± 9.3	71.8 ± 9.1	t = 0.69	0.493
Sex, n (%)	Male	39 (76.5%)	36 (78.3%)	75 (77.3%)	χ² = 0.13	0.715
	Female	12 (23.5%)	10 (21.7%)	22 (22.7%)		
Risk stratification, n (%)	Low	28 (54.9%)	22 (47.8%)	50 (51.5%)	χ² = 1.84	0.398
	Intermediate	21 (41.2%)	20 (43.5%)	41 (42.3%)		
	High	2 (3.9%)	4 (8.7%)	6 (6.2%)		
Number of prior recurrences, mean ± SD		1.8 ± 0.9	1.9 ± 1.0	1.85 ± 0.95	t = -0.49	0.625

**Table 2:** Comparison of recurrence rates between TULA and TURBT groups during initial follow-up cystoscopy Data are presented as n (%). Statistical significance was assessed using the chi-square (χ²) test. A p-value of <0.05 was considered statistically significant. TURBT: Transurethral resection of bladder tumour; TULA: Transurethral laser ablation

Outcome	TULA (n = 51)	TURBT (n = 46)	Total (n = 97)	Test statistic	P-value
Recurrence, n (%)	21 (41.2%)	19 (41.3%)	40 (41.2%)	χ² = 0.001	0.981
No recurrence, n (%)	30 (58.8%)	27 (58.7%)	57 (58.8%)	-	-

## Discussion

Our study shows that there was no statistically significant difference between TULA performed under local anaesthesia in an outpatient setting and TURBT in preventing early tumour recurrence. This aligns with multiple reports demonstrating that laser-based ablation achieves equivalent oncological outcomes without compromising safety [2-6].

Clinical effectiveness and safety

Laser ablation techniques confer clear perioperative advantages. Conventional TURBT carries risks such as obturator nerve reflex and bladder perforation, especially in lateral wall tumours, but meta-analyses and randomised controlled trials consistently report much lower complication rates with laser approaches [5,7,10]. Furthermore, laser resections provide higher-quality specimens with detrusor muscle, which is vital for accurate staging [7,10].

Patient-centred benefits

From the patient's perspective, TULA offers a streamlined experience. Avoiding general anaesthesia reduces perioperative risk, shortens recovery, and improves comfort. Long-term studies confirm that outpatient TULA is well tolerated, safe, and effective [6,11]. Cost analyses also demonstrate that TULA reduces healthcare expenditure by decreasing theatre time, admissions, and resource use [2,12].

Establishing an outpatient TULA service

**Figure 2:**
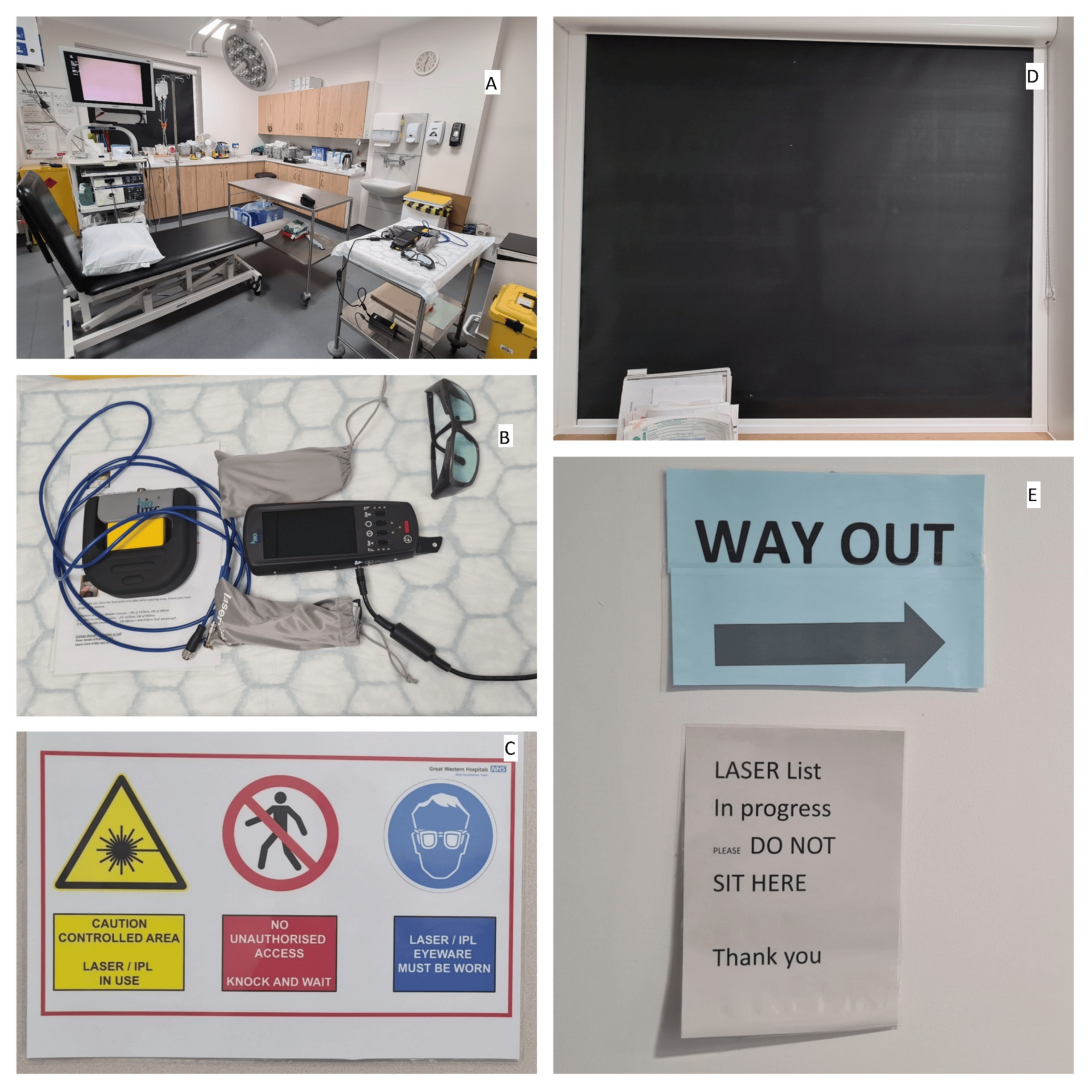
Cystoscopy clinic setup and laser safety measures (A) Cystoscopy clinic room with laser trolley positioned on the right. (B) Close-up of the laser trolley showing equipment and usage instructions. (The pictured equipment is the portable diode laser system (CeramOptec GmbH, Germany) currently used in the clinic for TULA procedures. Please note that this system was acquired post-study completion and was not utilised for the procedures or data collection presented in this retrospective analysis.) (C) Door signage for the cystoscopy clinic. (D) Installed protective blinds within the clinic to ensure laser safety. (E) Wall-mounted signage at the clinic entrance. TULA: Transurethral laser ablation; IPL: Intense pulsed light

A key contribution of this study is the detailed discussion of how such a service can be effectively implemented. Successful establishment requires several core components. First, all staff should complete formal laser safety training and competency assessments addressing both technical application and patient communication, with oversight provided by a designated laser protection supervisor and advisor in accordance with national safety regulations. Second, the clinical environment must be adapted to ensure safety, including the use of warning lights and signage, covered windows, designated treatment zones, mandatory laser eye protection for staff and patients, and integration of fire safety and smoke evacuation protocols (Figure 2). Third, implementation must be underpinned by a robust regulatory and governance framework, including hospital board approval, compliance with national legislation, standard operating procedures, regular auditing, and adequate indemnity cover. Appropriate patient selection is also essential, with ideal candidates being those with small, recurrent, low-grade NMIBC. Patients should be provided with clear pre-procedure information regarding expectations, recovery, and follow-up, and procedures must typically be performed under flexible cystoscopy with local anaesthetic gel to avoid the need for fasting or recovery delays. Finally, continuous audit and quality assurance are critical, with prospective monitoring of recurrence, complications, and patient satisfaction, which, in our experience, demonstrated oncological safety and facilitated acceptance by both staff and patients. Evidence from other centres supports these findings, showing that once established, TULA services enhance efficiency, improve patient satisfaction, and release operating theatre capacity [2,6,7].

Integration with emerging evidence

Recent studies strengthen the case for outpatient TULA. En bloc laser resection has been shown to reduce residual tumour rates and improve staging accuracy [13]. Randomised controlled trials demonstrate non-inferior recurrence-free survival at 1-2 years compared with TURBT [14]. Newer laser technologies, such as thulium and dual-wavelength diode systems, may further enhance cutting precision, haemostasis, and visualisation [9,15]. Meanwhile, enhanced cystoscopic imaging (e.g., blue-light or narrow-band imaging) improves detection of small recurrences and could complement laser treatment in future outpatient models [16].

Study limitations

The study design was retrospective, with a modest sample size and a short follow-up period, limiting the ability to draw conclusions about long-term outcomes, with insufficient time for recurrence assessment in the study. Additionally, enhanced cystoscopic techniques were not employed, which may have led to an underestimation of recurrence detection [16]. Moreover, patients in the TULA arm were selected based on their individual clinical assessments. Prospective multicentre trials with longer follow-up are warranted to validate these findings.

## Conclusions

In conclusion, TULA may represent a safe and effective alternative to conventional TURBT for the management of recurrent superficial bladder tumours. Its comparable efficacy to TURBT in preventing tumour recurrence underscores its potential as a reasonable alternative, especially for patients at higher risk of complications from general anaesthesia. However, this retrospective study is limited by a small sample size, short follow-up period, and the absence of enhanced cystoscopy, which may have led to an underestimation of recurrence rates. Additionally, patient selection for TULA was individualised. Prospective studies with larger sample sizes and longer follow-up are warranted to validate these results and provide more comprehensive evidence for informed clinical decision-making.

Moreover, the establishment of an outpatient TULA service requires a coordinated and comprehensive approach. By prioritising patient safety, operational efficiency, and quality of care, healthcare institutions can successfully implement and sustain such services, offering patients with recurrent superficial bladder tumours a safe, effective, and minimally invasive treatment option.
